# A critical overview of tools for assessing cognition in bipolar disorder

**DOI:** 10.1017/S2045796022000555

**Published:** 2022-10-03

**Authors:** Maria Gloria Rossetti, Francesca Girelli, Cinzia Perlini, Paolo Brambilla, Marcella Bellani

**Affiliations:** 1 Department of Neurosciences and Mental Health, Fondazione IRCCS Ca' Granda Ospedale Maggiore Policlinico, Milan, Italy; 2 UOC Psichiatria, Azienda Ospedaliera Universitaria Integrata, Verona, Italy; 3 Section of Clinical Psychology, Department of Neurosciences, Biomedicine and Movement Sciences, University of Verona, Verona, Italy; 4 Department of Pathophysiology and Transplantation, University of Milan, Milan, Italy; 5 Section of Psychiatry, Department of Neurosciences, Biomedicine and Movement Sciences, University of Verona, Verona, Italy

**Keywords:** Bipolar disorder, cognitive neuroscience, psychological assessment, rating scale

## Abstract

Cognitive deficits are prevalent in bipolar disorder even during the euthymic phase, having a negative impact on global functioning and quality of life. As such, more and more mental health professionals agree that neuropsychological assessment should be considered an essential component of the clinical management of bipolar patients. However, no gold standard tool has been established so far. According to bipolar disorder experts targeting cognition, appropriate cognitive tools should be brief, easy to administer, cost-effective and validated in the target population. In this commentary, we critically appraised the strengths and limitations of the tools most commonly used to assess cognitive functioning in bipolar patients, both for screening and diagnostic purposes.

There is growing consensus that cognitive impairment is a key feature of bipolar disorder (BD), visible even before the first manifestation of the disease and in both acute and remitted phases (Bora *et al*., 2010*a*; Bora and Pantelis, 2015). Cognitive deficits of bipolar patients primarily affect long-term verbal and visual memory skills, working memory, attention and psychomotor speed, executive functions and language (Kurtz and Gerraty, 2009; Baune *et al*., 2013). Most importantly, these deficits slow down the recovery and worsen educational and occupational attainment (Robinson *et al*., 2006; Baune *et al*., 2013), resulting in poorer psychosocial functioning and lower quality of life for the patients (Baune *et al*., 2013). Current evidence highlights the need to systematically implement neuropsychological assessment in the clinical management of BD as an essential component for planning tailored interventions. Consistently, increasing efforts have been made to develop standardised and validated instruments for assessing cognition in BD, although a gold standard tool has not been established yet(Bakkour *et al*., 2014).

In this commentary, we critically appraised the strengths and limitations of the tools most commonly used to assess cognition in BD, both for screening purposes or to detect cognitive impairment Fig. 1.

**Fig. 1.**
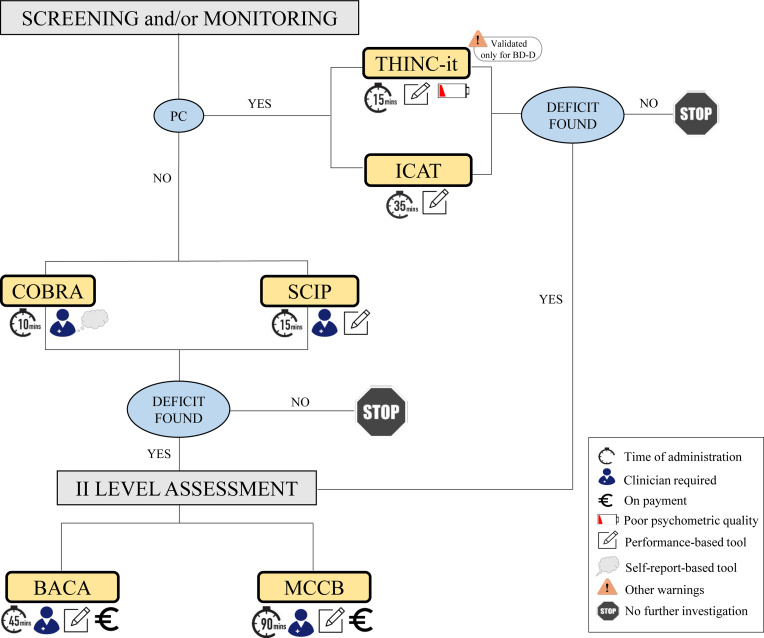
A diagram to help healthcare professionals to make the best use of tools commonly used to assess cognition in BD.

In 2018 the task force targeting cognition of the International Society for Bipolar Disorder (ISBD) published consensus-based recommendations on how to assess and manage cognitive impairment in BD (Miskowiak *et al*., 2018). The key recommendations were that mental health professionals formally screen cognition of bipolar patients whenever possible, by means of brief, cost-effective and easy-to-administer tools, and refer patients for extensive neuropsychological evaluation when clinically required. Specifically, the task force indicated the Cognitive Complaints in Bipolar Disorder Rating Assessment (COBRA) and the Screen for Cognitive Impairment in Psychiatry (SCIP) as the most feasible tools for the screening of subjective and objective cognition, respectively (Miskowiak *et al*., 2018). Both instruments are free of charge, brief, and do not require specific training for their administration.

The COBRA is a self-report rating scale specifically developed to test subjective cognition in BD. The instrument has high practical utility but has shown only small-to-moderate sensitivity and specificity for detecting objective cognitive impairment (Jensen *et al*., 2015). Therefore, its use is recommended only in combination with other screeners for objective cognition (Miskowiak *et al*., 2018).

The SCIP is the pencil-and-paper tool most commonly used for screening objective cognition in psychiatric disorders (Guilera *et al*., 2009; Rojo *et al*., 2010; Schmid *et al*., 2021). The test has three parallel versions making it suitable for monitoring performance over time. The SCIP has been validated in BD, showing good sensitivity and specificity for detecting objective cognitive impairment (Guilera *et al*., 2009; Rojo *et al*., 2010; Cuesta *et al*., 2011; Jensen *et al*., 2015). However, for this tool (as for other prospective cognitive screening tests), there is still a lack of normative data to determine clinically significant change over time at the individual level (Purdon and Psych, 2005).

A modified web-based version of the SCIP was developed in 2019, i.e. the Internet-Based Cognitive Assessment Tool (ICAT) (Hafiz *et al*., 2019; Miskowiak *et al*., 2021). The ICAT is a patient-administered test that allows the screening of objective cognitive deficits online, using gold-standard, performance-based cognitive tasks. The ICAT has shown good sensitivity to cognitive impairment of bipolar patients and high concurrent validity with the SCIP (Miskowiak *et al*., 2021). Overall, ICAT allows assessment and monitoring of patients' cognition at a much larger scale and at a reduced cost than paper-and-pencil tests. Nonetheless, the ability to operate computers/tablets independently and the presence of an internet connection are essential prerequisites for using this instrument.

Another web-based, patient-administered cognitive screening tool that can be potentially applied to bipolar patients is the THINC-IT. This test was released prior to ICAT but was originally designed for patients with unipolar depression (McIntyre *et al*., 2017). THINC-it employs gamified cognitive tasks to engage patients in taking the tests. Similar to ICAT, THINC-IT is free of charge, brief, and user-friendly; however, it lacks an assessment of verbal learning and memory, which (ideally) should always be present in screening tools for BD since impairments in this domain represent a core feature of the disorder that is common also during remitted states (Arts *et al*., 2008; Bora *et al*., 2010*b*) and contributes to poor occupational and daily functioning (Robinson *et al*., 2006; Baune *et al*., 2013). Of note, THIC-IT was tested on patients with BD-II only (Zhang *et al*., 2020). Therefore, further studies including both BD-I and BD-II are needed to verify the suitability and sensitivity of this battery for cognitive assessment in BD.

Overall, cognitive screeners for use in BD take little time to administer, are easy to use and cost-effective and appear feasible in the clinical management of BD. However, they do not measure real-life functions and cannot replace a thorough neuropsychological evaluation (Miskowiak *et al*., 2018).

As for comprehensive neuropsychological assessments, the MATRICS Consensus Cognitive Battery (MCCB) and the Brief Assessment of Cognition In Affective Disorders (BAC-A) are among the most commonly used tools with bipolar patients (Yatham *et al*., 2010; Van Rheenen and Rossell, 2014).

The MCCB was developed originally for schizophrenia (Nuechterlein *et al*., 2008). Then, the cognition task force of the ISBD endorsed the applicability and clinical utility of most MCCB subtests for use in BD, further recommending the inclusion of more complex measures of verbal learning or executive function (Yatham *et al*., 2010). Preliminary evidence has shown good sensitivity of the MCCB in distinguishing between the cognitive functioning of bipolar and control groups and a need for a more thorough evaluation of specific domains as suggested by the ISBD (Van Rheenen and Rossell, 2014).

The BAC-A has been designed specifically for use in affective disorders (Keefe *et al*., 2014). The battery includes eight subtests measuring both non-affective and affective cognition. Moreover, it is suitable for test-retest evaluations as the verbal tests include alternative forms. Recent studies have demonstrated that the BAC-A is sensitive to the cognitive impairments of patients with BD in both affective and non-affective cognitive domains and has good test-retest reliability (Keefe *et al*., 2014; Bauer *et al*., 2015; Barbosa *et al*., 2018; Lee *et al*., 2018; Rossetti *et al*., 2022).

Overall, evidence suggests that both the MCCB and the BAC-A have proved sensitive to the cognitive impairment of bipolar patients and may be used for diagnostic purposes. However, these tools are expensive, time-consuming and require extensive training for their administration. Such factors limit their applicability in clinical practice.

In this commentary, we critically appraised the strengths and limitations of the tools most commonly used to assess cognition in BD (Table 1). What emerges is that there are no tools more appropriate than others *per se*, as the suitability and clinical applicability of these instruments may depend on multiple factors. Among others, the target population (e.g. young vs elderly patients; people able vs not able to operate a computer); the purpose of the evaluation (e.g. cognitive screening vs exhaustive diagnostic evaluation; single evaluation vs monitoring over time); the characteristics of the tool itself (e.g., self-report vs performance-based; paper-and-pencil vs computerized; free of charge vs on payment); (iv) the clinical setting (e.g., presence of qualified vs unqualified staff; time and space resources). Based on these factors, we propose a diagram that may help healthcare professionals to make the best use of the instruments described above. The diagram visually summarises both the characteristics of each tool and the cognitive assessment process. As regards the properties of the tools, we used icons under each tool name (and listed in the caption) which refer to administration time, the need for the clinician, the psychometric property of the instrument, whether the tool is performance- or self-report-based and, finally, if it is on payment. As for the assessment process, the clinician is first asked to screen and/or monitor (“screening and/or monitoring” in the upper part of the schema) the patient's cognitive performance using one of four instruments (THINC-it, ICAT and COBRA and SCIP) based on the need for a computer (i.e., PC yes/no). If deficits are found at this point, the healthcare professional can proceed to a II-level assessment, choosing between BACA and MCCB. Otherwise, a stop icon warns the clinician to interrupt the evaluation. We acknowledge that this diagram is a preliminary proposal deriving from our clinical and research experience with BD patients. Thus, it would need to be further studied and possibly endorsed by other research groups.

**Table 1.** Characteristics, strengths and limitations of tools commonly used for cognitive assessment in BD

Tool	Characteristics	Strengths	Limitations
**COBRA**	The COBRA is a 16-item self-report instrument that allows measuring subjective cognitive difficulties including executive function, processing speed, working memory, verbal learning and memory, attention and concentration and mental tracking. All items are rated using a 4-point scale: 0 = never, 1 = sometimes, 2 = often and 3 = always. The total score is obtained by adding up the scores of every item. The higher the score, the higher the subjective complaints. Available at: https://www.isbd.org/Cognitive-Assessments	Free of charge Brief (≈10 min) and easy-to-administer High practical utility Can be administered after minimal training Available in many languages Specifically designed for BD population	Self-report rating scale Suboptimal sensitivity/specificity for objective cognitive impairment Can be used for screening purposes only
**SCIP**	The SCIP is a brief, easy-to-administer objective screening tool requiring only a pencil and a test sheet, with an administration time of approximately 15 min. It was designed to detect cognitive deficits in psychiatric populations using t verbal learning tests (immediate and delayed), a working memory test, a Verbal Fluency Test, and a processing speed test. Exists in three alternative forms. Available at: https://www.isbd.org/SCIP-registration	Free of charge Brief (≈15 min) and easy-to-administer High practical utility Can be administered after minimal training Evaluate objective (performance-based) cognition Available in many languages Allows for repeated testing over time	Not specifically designed for BD Can be used for screening purposes only
**THINC-it**	Web-based screening tool originally developed to assess cognition in MDD. It measures both subjective and objective cognition by employing four objective tests (attention & executive functions, working memory and processing speed) and one self-report questionnaire (PDQ-5-D) measuring attention and concentration, prospective memory, retrospective memory, planning, and organisation. Available at: https://progress.im/en/content/download-thinc-it%C2%AE-tool	Free of charge Brief (≈15 min) and user-friendly Can be used remotely (patient-administered) Evaluate both objective (performance-based) and subjective cognition Available in 15 languages	Unmonitored assessment setting Not specifically designed for BD Absence of tests for the evaluation of ‘core’ cognitive domains for BD Subjects must be able to operate a computer/tablet independently Requires internet connection Cannot be used for diagnostic purposes
**ICAT**	Web-based cognitive test adapted from SCIP. It includes five subtests that evaluate 4 cognitive domains: verbal learning, working memory, delayed verbal learning and psychomotor speed. Available at: https://icat.cachet.dk/	Free of charge Brief (≈35 min) and user-friendly High practical utility Can be used remotely (patient-administered) Evaluate objective (performance-based) Cognition	Unmonitored assessment setting Not specifically designed for BD Available only in English and Danish Subjects must be able to operate a PC independently Requires internet connection Cannot be used for diagnostic proposes
**MCCB**	Originally developed to assess cognition in SCZ. It evaluates seven cognitive domains: processing speed, attention/vigilance, working memory, verbal learning, visual learning, reasoning and problem-solving, and social cognition. Available at: https://www.matricsinc.org/how-to-order/	Assess objective (performance-based) cognition Can be used for full diagnostic evaluation Comprises tests previously validated and widely used	Not specifically designed for BD Time-consuming (≈90 min) Expensive (>1000 $) Low practical utility Requires extensive training to be used
**BACA**	The BAC-A is designed to assess cognitive functions in Affective Disorders. The battery lasts approximately 45 min and comprises eight tasks evaluating visuomotor abilities, working memory, learning and declarative memory, attention, verbal fluency, problem-solving, affective interference and affective inhibition. Available at: https://verasci.com/what-we-do/endpoints-assessments/bac/	Assess objective (performance-based) cognition Includes tasks of affective cognition Validated in various languages Can be used for full diagnostic evaluation Allows for repeated testing over time	Time-consuming (≈45 min) Expensive (≈1000 $) Requires extensive training to be used

BACA, Brief Assessment of Cognition in Affective Disorder; BD, Bipolar Disorder; COBRA, Cognitive Complaints in Bipolar Disorder Rating Assessment; ICAT, Internet Based Cognitive Assessment Tool; MCCB, MATRICS Consensus Cognitive Battery; PDQ-5-D, Perceived Deficits Questionnaire for Depression; SCIP, Screen for Cognitive Impairment in Psychiatry; SCZ, Schizophrenia; THINC-it, THINC-integrated tool.

## Data

All data used to write this paper is in the reference list.
